# Genome-wide identification, classification and expression analysis of the JmjC domain-containing histone demethylase gene family in maize

**DOI:** 10.1186/s12864-019-5633-1

**Published:** 2019-04-01

**Authors:** Yexiong Qian, Changle Chen, Lingyu Jiang, Jing Zhang, Qiaoyu Ren

**Affiliations:** Anhui Provincial Key Laboratory of the Conservation and Exploitation of Biological Resources, Anhui Normal University, Wuhu, 241000 China

**Keywords:** Epigenetics, Histone demethylation, *JHDM* gene family, JmjC domain, Heat stress, *Zea mays L*

## Abstract

**Background:**

Histone methylation mainly occurs on the lysine residues and plays a crucial role during flowering and stress responses of plants, through changing the methylation status or ratio of lysine residues. Histone lysine residues of plants can arise in three forms of methylation (single, double and triple) and the corresponding demethylation can also ensue on certain occasions, by which the plants can accommodate the homeostasis of histone methylation by means of lysine methyltransferase and demethylase. The JmjC domain-containing proteins, an important family of histone lysine demethylases, play a vital role in maintaining homeostasis of histone methylation in vivo.

**Results:**

In this study, we have identified 19 JmjC domain-containing histone demethylase (JHDM) proteins in maize. Based on structural characteristics and a comparison of phylogenetic relationships of *JHDM* gene families from *Arabidopsis*, rice and maize, all 19 JHDM proteins in maize were categorized into three different subfamilies. Furthermore, chromosome location and schematic structure revealed an unevenly distribution on chromosomes and structure features of *ZmJMJ* genes in maize, respectively. Eventually, the 19 *ZmJMJ* genes displayed different expression patterns at diverse developmental stages of maize based on transcriptome analysis. Further, quantitative real-time PCR analysis showed that all 19 *ZmJMJ* genes were responsive to heat stress treatment, suggesting their potential roles in heat stress response.

**Conclusions:**

Overall, our study will serve to present an important theoretical basis for future functional verification of *JHDM* genes to further unravel the mechanisms of epigenetic regulation in plants.

**Electronic supplementary material:**

The online version of this article (10.1186/s12864-019-5633-1) contains supplementary material, which is available to authorized users.

## Background

In eukaryotic genomes, the histones (H2A, H2B, H3 and H4) wind DNA into nucleosome, and then the DNA-protein complexes are further compressed to form a complex structure known as chromatin. The core nucleosome is composed of histone octamer (containing two copies of each of the four histone proteins H2A, H2B, H3 and H4), and its tail has been elucidated to have posttranslational modifications [1, 2] including methylation, acetylation, phosphorylation, ADP-ribosylation and ubiquitination, which are designated as the histone code. These histone modifications not only affect and change the status of genomic chromatin, but also regulate the transcriptional process of gene expression [3]. Therefore, these histone modifications, such as methylation and demethylation, have become one of the hot topics in epigenetic research in recent years.

Histone methylation and demethylation play crucial roles in regulating gene expression, genome integrity, and epigenetic inheritance [4–7]. Histone methylation can occur at various lysine and arginine residues, including K4, K9, K27, K36, and K79 in histone H3 and K20 in histone H4 [4, 8]. Some protein families including PRMT and SET domains are responsible for histone methylation of specific arginine and lysine residues and have been characterized, respectively. Moreover, two kinds of histone demethylase are known to play important roles in demethylation of lysine residues in organisms [4]. Based on the different ways of their action, these lysine demethylases are sorted into two categories: (1) LSDs, lysine-specific demethylases 1, remove the methyl groups by amine oxidation; (2) JHDMs (JmjC domain-containing histone demethylases), the demethylases containing the JmjC domains, remove the methyl groups through hydroxylation [9, 10]. These two types of enzymes require various cofactors and act on particular substrates. For instance, LSD1 relies on the involvement of Flavin adenine dinucleotide (FAD), which acts only on the mono- and di-methylated lysines. However, JHDM resorts to the participation of Fe and α-ketoglutarate (α-KG), which can act on the methylation of three forms of lysine [6, 11, 12].

The JmjC domain-containing proteins in plants have been demonstrated to be involved in regulating epigenetic processes and in plant growth and development [4, 7, 13]. Many members of the *JmjC* gene family from various plant species have been identified and characterized in plants. For instance, *Arabidopsis* contained 21 JmjC proteins, which are involved in the regulation of RNA silencing, DNA methylation, Brassinosteroid (BR) signaling pathway, affecting flowering, and biorhythm and bud regeneration and are subdivided into five groups: JARID1/KDM5, JHDM3/KDM4, JHDM2/KDM3, JHDM6 and a class of proteins just containing the JmjC domain [10]. In the JARID1/KDM5 group, there are a couple of *Arabidopsis JmjC* genes (*AtJMJ14* and *AtJMJ15*) encoding histone H3K4 demethylases, which are capable of reversing H3K4me3, H3K4me2, and H3K4me1 in vivo and regulate flowering time in *Arabidopsis* [14]. In the JHDM3/KDM4 group, AtJMJ11/ELF6 (EARLY FLOWERING 6) and its close homologue, AtJMJ12/REF6 (RELATIVE OF EARLY FLOWERING 6) have been shown to play divergent roles in flowering time control. Loss-of-function mutation of AtJMJ11/REF6 leads to increased expression of the flowering repressor FLC (FLOWERING LOCUS C) and hence late flowering [15]. In addition, AtJMJ11/ELF6 and AtJMJ12/REF6 are revealed to interact with BES1, a transcription factor in the brassinosteroid (BR) signaling pathway [16], indicating that histone demethylases can be recruited to target genes by interacting with sequence specific transcription factors. In the JHDM2/KDM3 group, the histone H3K9 demethylase, AtJMJ25/IBM1 (INCREASE IN BONSAI METHYLATION 1) protects genes from CHG (H represents A, T, or G) hypermethylation by CMT3 (CHROMOMETHYLASE 3) [17, 18]. Mutations in AtJMJ25/IBM1 result in increased DNA methylation and a variety of developmental phenotypes that rely on histone H3K9 methylation and can be completely inhibited by mutations in KYP [19, 20]. Furthermore, in the JHDM6 and a class of proteins just containing the JmjC domain groups, AtJMJ30/JMJD5, an evening-expressed gene, is the sole AtJMJ protein to exhibit a robust circadian rhythm of expression [21–24]. By analysis of loss- and gain-of-function mutants, the function of AtJMJ30 has been verified as a genetic regulator of period length in the *Arabidopsis* circadian clock [21].

Maize (*Zea mays L.*), as one of the most important crop species in the world, has become one of the important model monocot species for functional genomics analysis. It has been of great significance to the study of molecular biology in plants. However, little is known about the identification and function of histone demethylase gene family in maize. In this study, we performed a comprehensive analysis of *ZmJMJ* genes, including their phylogenetic relationships, gene structure, domain architecture, chromosome location, duplication patterns, and expression profiles, which will facilitate future studies to elucidate the exact biological functions of the *ZmJMJ* genes in maize.

## Results

### Identification of the members of *JHDM* gene family in maize

To identify all possible homologs of *JHDM* gene family in maize, the amino acid sequences of *Arabidopsis* and rice JmjC domain-containing proteins and their JmjC domains (Pfam: PF02373, SMART: SM00558) were adopted as the query sequences to search against the maize genome database with BlastP program. Subsequently, the candidates were surveyed using Pfam and SMART databases to further verify the presence of the JmjC domains, respectively. Consequently, 19 non-redundant *JHDM* genes were identified and described from the original data after removing the redundant sequences. The total number of *JHDM* genes identified in maize (19) is similar to that in either *Arabidopsis* (21) or rice (20) (Table 1). We aligned all the *ZmJMJ* genes with *Arabidopsis* and rice *JHDM* genes to generate a phylogenetic tree for classification of maize *JHDM* genes. Based on their relationships with *Arabidopsis* and rice *JHDM* genes, the 19 *ZmJMJ* genes were divided into three distinct subfamilies, including the *JARID1/KDM5* subfamily (8 genes), the *JHDM3/KDM4* subfamily (8 genes) and the *JHDM2/KDM3* subfamily (3 genes) (Table 1). A total of 19 *JHDM* genes are uniformly named as *ZmJMJ*1-*ZmJMJ*19 based on the position of their corresponding genes on chromosomes 1–10 (Additional file 1: Table S1). Then, the filtered maize protein sequences were analyzed by Expasy (https://web.expasy.org/compute_pi/) to further confirm some physical parameters including the number of amino acid residues, the isoelectric point and the molecular weight and so on. The length of the *ZmJMJ* open reading frames (ORFs) varied from 1500 bp for *ZmJMJ1* to 4569 bp for *ZmJMJ18*, with the respective coding potential of 499 and 1522 amino acids (Additional file 1: Table S1). Furthermore, the molecular weight varied from 56,430.79 to 167,759.67, indicating that ZmJMJ proteins share a variety of molecular weight scope.

**Table 1:** *JHDM* gene distribution among species that were used in this study

Category (Genome size)	Maize (2300Mbp)	*Arabidopsis* (125Mbp)	Rice (389Mbp)
*JARID1*	8	6	4
*JHDM3*	8	3	5
*JHDM2*	3	6	4
*JHDM6*	0	3	2
*JmiC-only*	0	3	5
Total number	19	21	20

### The structures of identified JHDM proteins in maize

**Fig. 1:**
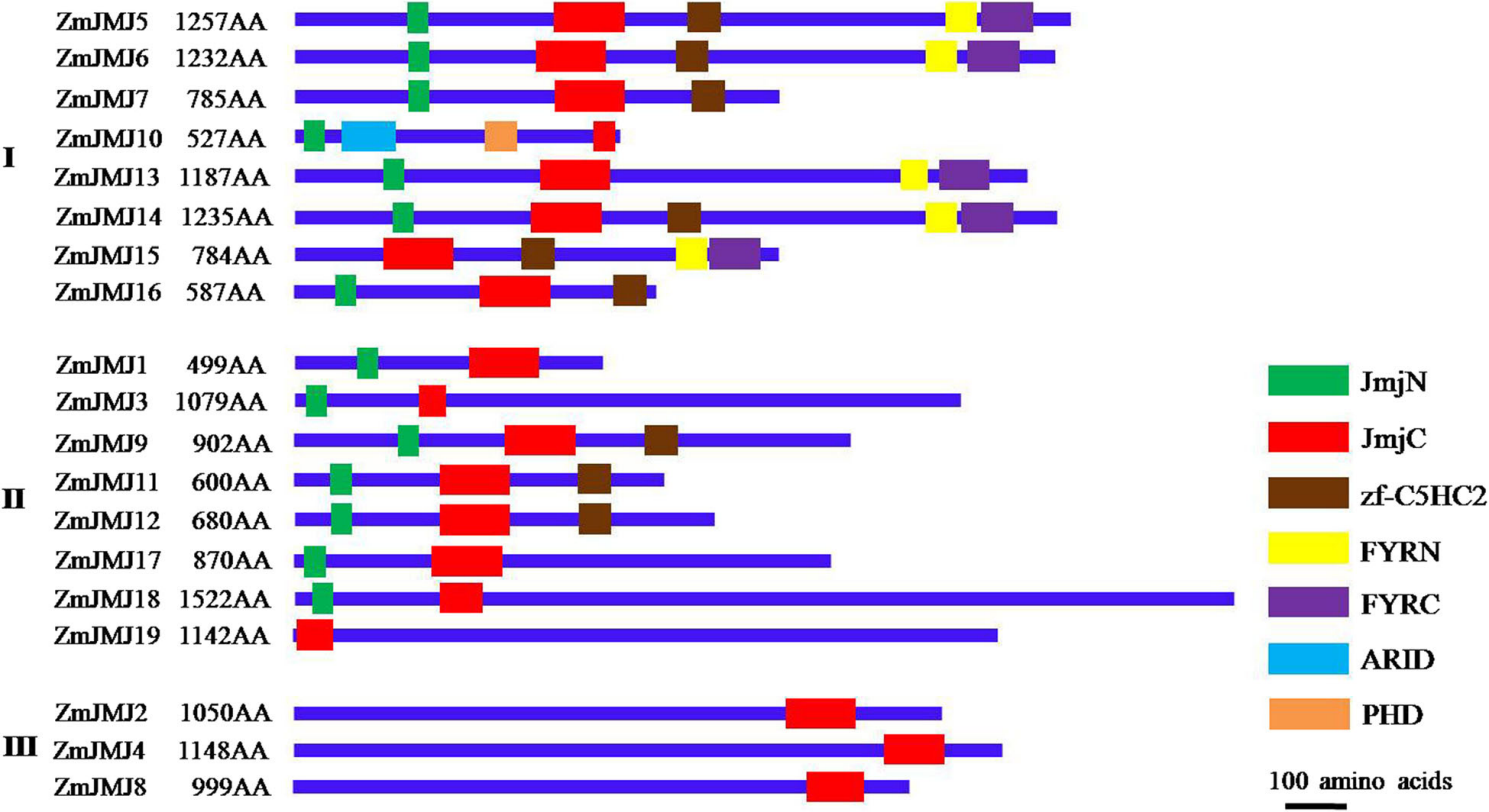
Schematic structure of JHDM proteins in maize. Schematic representation of conserved domains identified among each subfamily of maize JHDM proteins. The protein is represented as a blue box; the N terminus is on the left for each representation. The location and size of domains are shown by different color rectangles as indicated in the key. JmjN, Jumonji N domain; JmjC, Jumonji C domain; zf-C5HC2, Zinc finger of C5HC2-type; FYRN,“FY-rich” domain N-terminal; FYRC, “FY-rich” domain C-terminal; ARID, AT-rich interaction domain; PHD, plant home box domain

To gain more insights into the diversity of the domain architecture, the structure types of 19 maize JHDM proteins were determined by Pfam (http://pfam.xfam.org/) and SMART (http://smart.embl-heidelberg.de/) with default parameters. Based on the phylogenetic analysis of the diverse structural characteristics of *Arabidopsis* JHDM protein subfamilies [11], the maize JHDM protein family were mainly categorized into three subfamilies: subfamily I(JARID1/KDM5), subfamily II(JHDM3/KDM4) and subfamily III (JHDM2/KDM3), with each subfamily containing 8, 8 and 3 members, respectively (Fig. 1). In JARID1/KDM5 subfamily, the majority of members share five diverse conserved domains including JmjN, JmjC, zf-C5HC2, FYRN and FYRC domains, while ZmJMJ10 contains additional PHD and ARID domains. However, in JHDM2/KDM3 subfamily or JHDM3/KDM4 subfamily, there is only one JmjC domain or three diverse conserved domains, respectively. Among the three subfamilies, all of these proteins all share a conserved JmjC domain, which is characteristic of JHDM proteins and related to the demethylation process of the histone lysine site of action. In addition, the JmjN domain is the second most widespread domain, appearing in the majority of members of two groups, the JARID1/KDM5 and JHDM3/KDM4 subfamilies. The zf-C5HC2 domain was also identified in the JARID1/KDM5 and JHDM3/KDM4 subfamilies. However, there are still some members of the JARID1/KDM5 and JHDM3/KDM4 subfamilies not displaying the zf-C5HC2 domains. It still remains a possibility that the zf-C5HC2 domains in these proteins are lower conserved and hard to be detected under current analysis parameters. Five of members of the JARID1/KDM5 subfamily contain FYRN and FYRC domains, which may hold chromatin-binding activity [11] or contribute to JmjC domain function by collaborating with other proteins.

Furthermore, two important cofactors, Fe (II) iron and α-KG, have been verified to be necessary to the JmjC domain-containing proteins to perform their demethylase activity [25, 26]. A total of five conserved amino acid residues, namely three conserved residues (His188, Glu/Asp190, and His276) for the Fe (II) cofactor and other two conserved residues (Thr/Phe185 and Lys206) for α-KG are indispensable to bind these cofactors. To further confirm whether these conserved residues bound by cofactors had diverged among the ZmJMJ proteins, we aligned the domain sequences of the JmjC domain-containing proteins from *Arabidopsis*, rice and maize. On basis of the alignments and conserved amino acid residues, these proteins were sorted into two groups. The first group (JARID1/KDM5, JHDM3/KDM4), contains the three conserved amino acid residues, namely His (H), Glu (E) and His (H) for Fe (II) binding, and other two conserved amino acid ones, namely Phe (F) and Lys (K) for α-KG binding (Additional file 2: Figure S1, and Additional file 3: Figure S2), while the second group (JHDM2/KDM3) comprises the corresponding conserved residues, namely His (H), Asp (D) and His (H) for Fe(II) binding and Thr (T) and Lys (K) for α-KG binding (Additional file 4: Figure S3). Both of groups are compatible with lysine demethylation activity [11]. In short, the majority of members were detected to comprise these conserved amino acid residues bound by cofactors, although there are some special cases. For instance, the third site to interact with Fe (II) iron is missing in ZmJMJ10 protein, while the ZmJMJ19 protein only retains the third conserved His (H) residue for Fe (II) binding and is lack of other conserved sites for interacting with cofactors. Taken together, these conserved residues could play a vital role in involving demethylase activity of the JHDM protein family.

### The structures of identified *JHDM* genes in maize

**Fig. 2:**
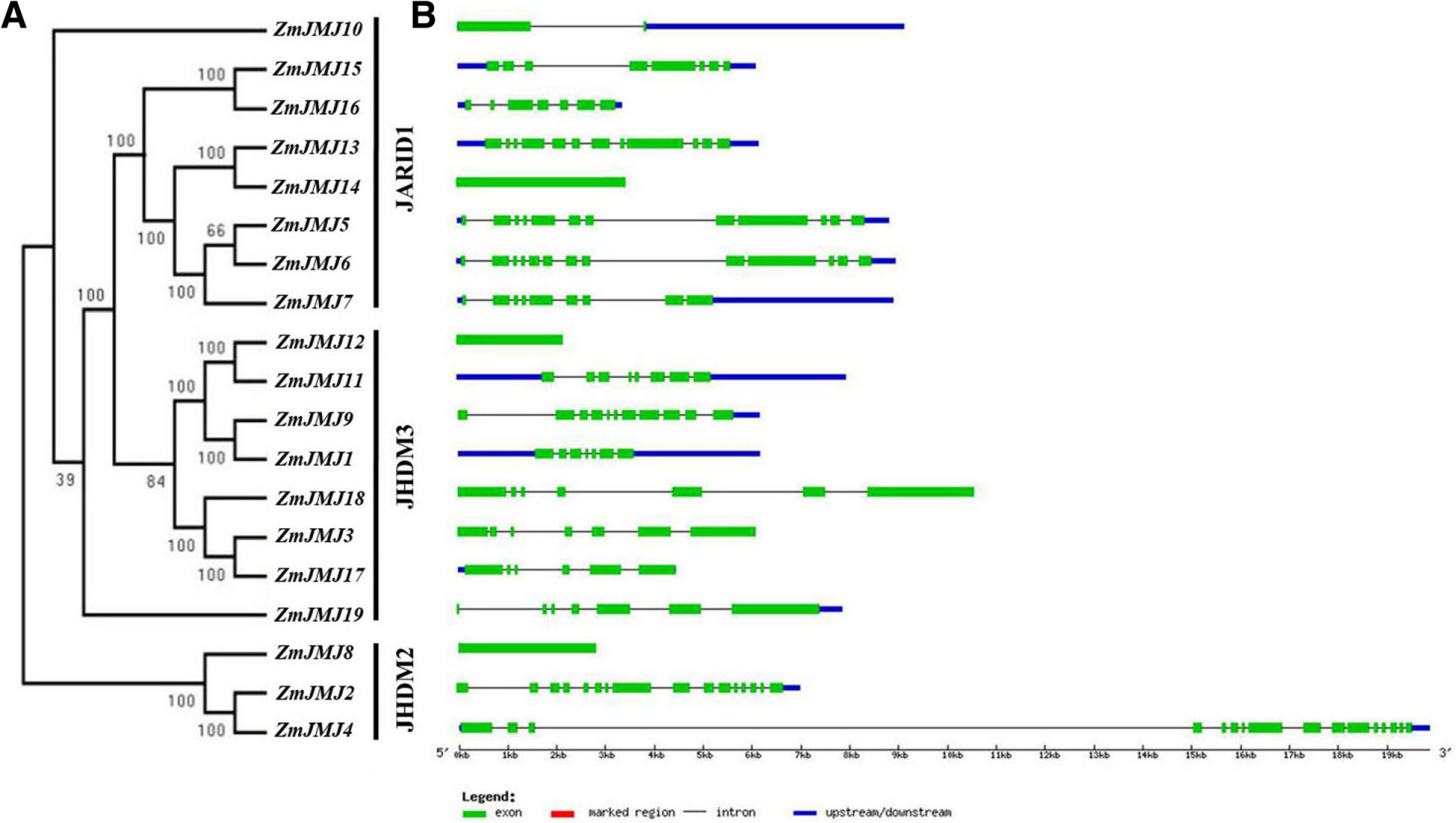
Phylogenetic relationship and gene structure of *JHDM* genes in maize. **a** The unrooted tree was constructed with MEGA6.0 program using the full-length sequences of the 19 maize *JHDM* genes. The bootstrap values are indicated at the branches in black numbers, and the genes were named according to their gene codes. **b** Exons and introns are indicated by white green boxes and single lines, respectively. Thick blue lines represent the untranslated regions (UTRs). The length of each *JHDM* gene can be estimated using the scale at the bottom

To illustrate the exon-intron structures of individual maize *JHDM* genes, comparison of the genomic sequences and their corresponding coding sequences (CDS) was operated using GSDS (Gene Structure Display Server) (http://gsds.cbi.pku.edu.cn/). The unrooted tree categorized the *JHDM* genes into three major groups with well-supported bootstrap values, indicating that the classification was reliable. The schematic structures clearly revealed that most of the paralogs share a similar exon-intron structure except *ZmJMJ8, ZmJMJ10, ZmJMJ12* and *ZmJMJ14* has only one exon, respectively (Fig. 2). In JARID1 subfamily, the most coding sequences are disrupted by approximate 7 to 12 exons except *ZmJMJ10* and *ZmJMJ14*. The *ZmJMJ2* and *ZmJMJ4* in JHDM2 subfamily have the largest number of exon or intron, and share a similar gene structure. Moreover, there was some deference in intron lengths and in the 5’UTR region between *ZmJMJ2* and *ZmJMJ4*, which might be related to the regulation of expression [4].

### Phylogenetic analysis of maize JHDM proteins

**Fig. 3:**
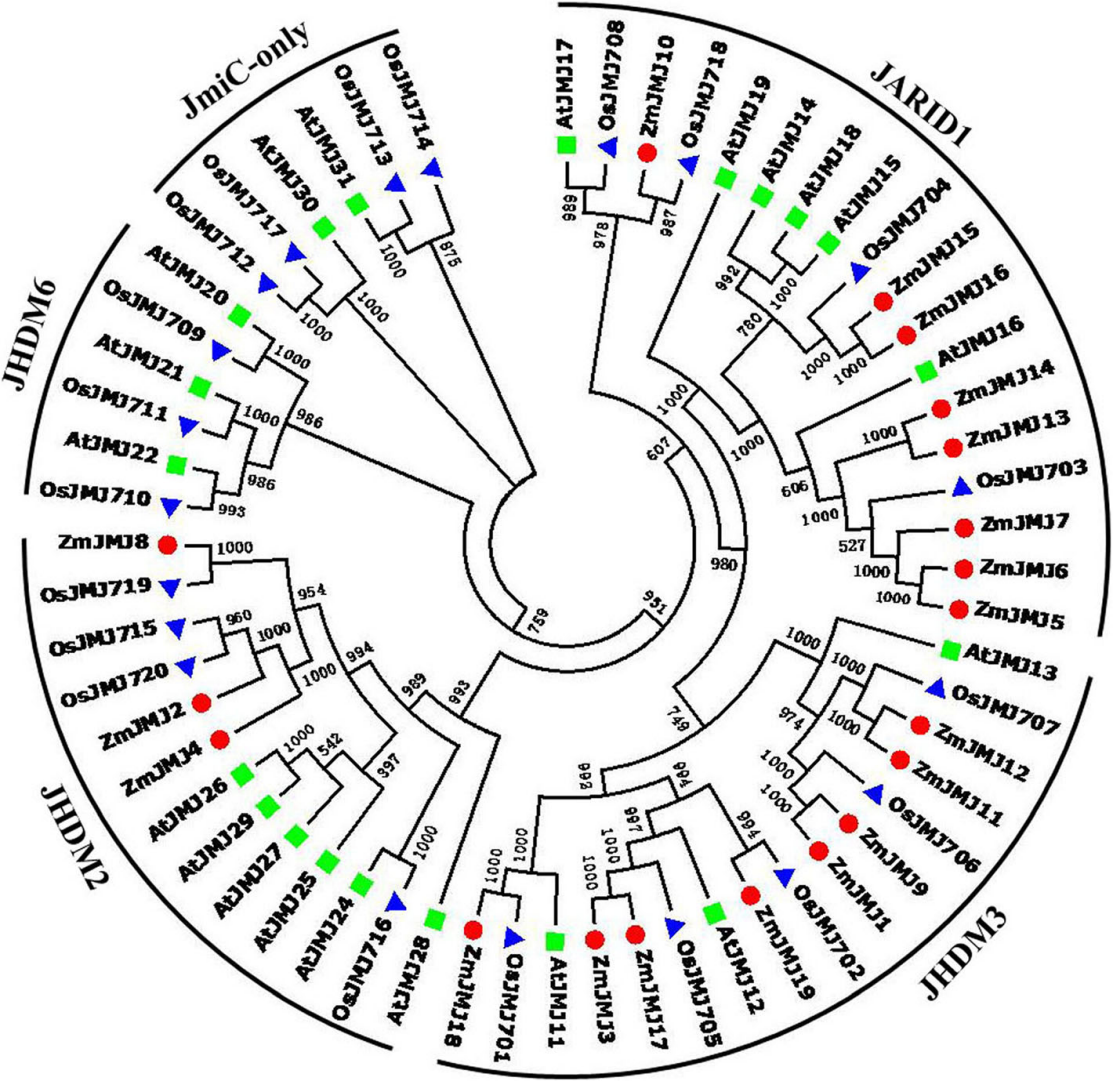
Phylogenetic analysis among JHDM proteins of maize, rice and *Arabidopsis*. Unrooted neighbor-joining (NJ) phylogenetic tree of maize, rice and *Arabidopsis* JHDM proteins with bootstrap values shown for each clade. The maize ZmJMJs have been highlighted for each group. Five clades are marked as JARID1, JHDM3, JHDM2, JHDM6 and JmjC-only. Protein sequences were downloaded from National Center for Biotechnology Information (NCBI)

To better discern the phylogenetic relationship among JHDMs in plants, and assess the evolutionary history of these protein families among maize, rice and *Arabidopsis*, we performed multiple sequence alignment with MEGA6.0 by using the full-length sequences of each JHDM protein from these plants and constructed an unrooted phylogenetic tree by using Neighbor-Joining method (Fig. 3).

The unrooted neighbor-joining phylogenetic tree was generated from aligned full-length protein sequences of 21 AtJMJs, 20 OsJMJs, and 19 ZmJMJs. Based on the comparison and analysis of JHDM diversity and phylogeny, these proteins were categorized into five major subfamilies: JARID1, JHDM3, JHDM2, JHDM6 and a class of proteins just containing the JmjC domain. However, the maize JHDM proteins were only divided into three subfamilies: JARID1, JHDM3, and JHDM2, with each subfamily containing 8, 8 and 3 members, respectively (Fig. 6). In JARID1 subfamily, maize ZmJMJ10 exhibits high homology with rice OsJMJ708 (E = 0.0), OsJMJ718 (E = 0.0) and *Arabidopsis* AtJMJ17 (E = 3e-161). Maize ZmJMJ14, ZmJMJ13, ZmJMJ7, ZmJMJ6, ZmJMJ5 share great similarity on evolution with *Arabidopsis* AtJMJ16 (E = 0.0; 0.0; 0.0; 0.0; 0.0) and rice OsJMJ703 (E = 0.0; 0.0; 0.0; 0.0; 0.0), and therefore they belong to orthologous proteins. In JHDM3 subfamily, maize ZmJMJ12, ZmJMJ11and rice OsJMJ707 (E = 0.0; 0.0) belongs to orthologous proteins. ZmJMJ9 and ZmJMJ1 share similarity with rice OsJMJ706 (E = 0.0; 0.0); and maize ZmJMJ19, ZmJMJ17 and ZmJMJ3 have the high similarity with *Arabidopsis* AtJMJ12 (E = 1e-71; 0.0; 2e-132) and rice OsJMJ705 (E = 9e-76; 0.0; 0.0) and OsJMJ702 (E = 0.0; 0.0; 1e-123). In JHDM2 subfamily, maize ZmJMJ8 and rice OsJMJ719 (E = 0.0) belong to orthologous proteins; and maize ZmJMJ2 and ZmJMJ4 share high similarity with rice OsJMJ715 (E = 0.0; 0.0) and OsJMJ720 (E = 0.0; 0.0).

### Chromosomal location and gene duplication of maize *JHDM* gene family

**Fig. 4:**
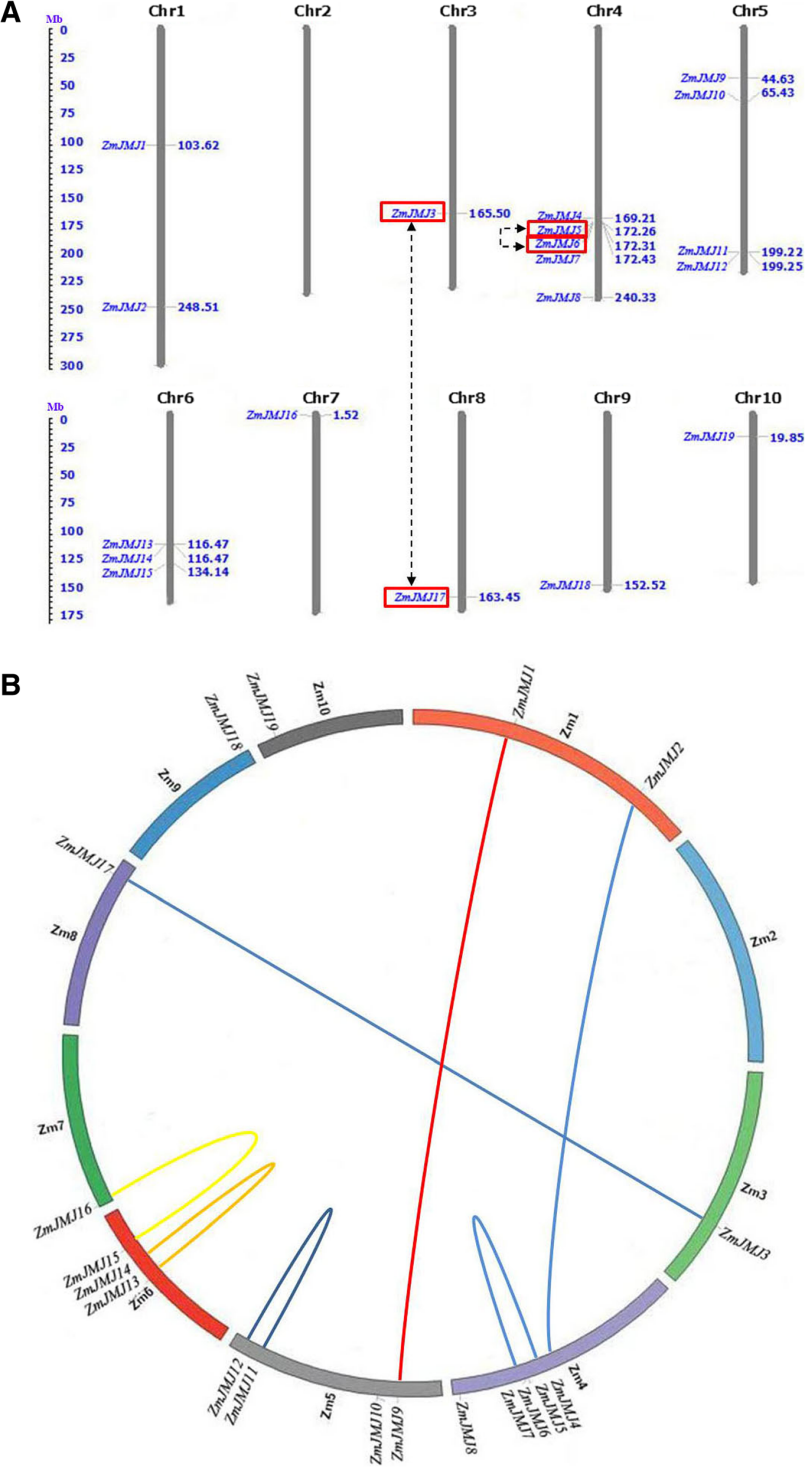
Chromosomal localization and gene duplication events of maize *JHDM* genes. **a** Distribution of *ZmJMJ* genes in maize chromosomes. Nineteen *ZmJMJ* genes were mapped on the nine maize chromosomes. The chromosome numbers are indicated at the top of each vertical gray bar. The gene names on the left side of each chromosome correspond to the approximate locations of each *ZmJMJ* genes. Both segmentally duplicated paralogs and tandemly duplicated homologs are connected and marked by dashed arrows. The scale on the left is in megabases. **b** Duplicated blocks in maize chromosomes. The circular image retrieved from PLAZA database show inter-chromosome homologous regions connected by bands in different colors. The chromosome numbers and *ZmJMJ* genes are indicated outside

The physical locations of *JHDM* genes in maize were investigated by analysis of genomic distribution on chromosomes. The 19 *ZmJMJ* genes were distributed unevenly across nine of all the ten chromosomes in the maize genome (Fig. 4). A great majority of *ZmJMJs* are clustered in the bottom of chromosomes and mainly distributed on three (chromosomes 4, 5, 6) out of nine maize chromosomes, which contained the largest number of *JHDM* gene family members with total12 genes, whereas the least number was detected on chromosomes 3, 7, 8, 9 and 10, containing only one *JHDM* gene, respectively (Fig. 4). Moreover, gene duplication events were surveyed to explore the evolutionary patterns of the *JHDM* gene family in maize. Analysis of maize *JHDM* genes in SyMAP [27] and PLAZA [28] revealed two *JHDM* genes (*ZmJMJ3* and *ZmJMJ17*) could be assigned to maize segmental duplication and another two *JHDM* genes (*ZmJMJ5* and *ZmJMJ6*) could be involved in tandem duplication (Fig. 4). These two gene pairs (*ZmJMJ5/ZmJMJ6* and *ZmJMJ3/ZmJMJ17*) were revealed to have very high homology in the sequences by analyses of sequence alignment, indicating that gene duplication events might have occurred during their process of evolution. Moreover, the duplicated genes were inconsistent with those identified in the phylogenetic tree because two *ZmJMJ* genes (*ZmJMJ15* and *ZmJMJ16*) were not in homologous regions.

### The cis-acting regulatory elements in the promoter of maize *JHDM* genes

**Fig. 5:**
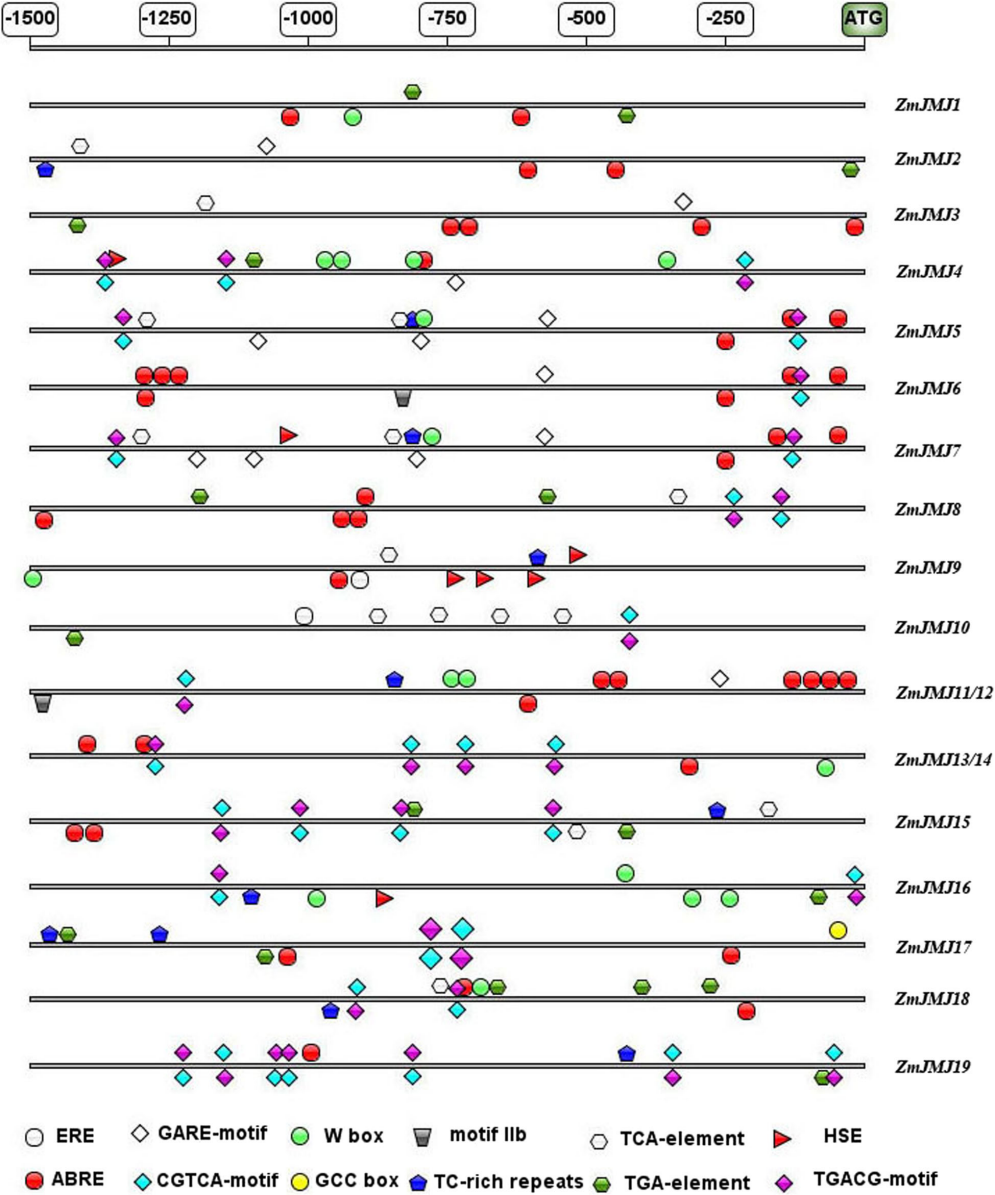
Distribution of major stress-related *cis*-elements in the promoter sequences of the 19 *ZmJMJ* genes. Putative ERE, ABRE, GARE-motif, CGTCA-motif, W box, GCC box, motif IIb, TC-rich repeats, TCA-element, TGA-element, HSE and TGACG-motif core sequences are represented by different symbols as indicated in figure key at the bottom. The cis-elements distributed on the sense strand and reverse strand are indicated above and below the black lines, respectively. ERE: ethylene-responsive element; ABRE: cis-acting element involved in the abscisic acid responsiveness; GARE-motif: gibberellin-responsive element; CGTCA-motif: cis-acting regulatory element involved in the MeJA-responsiveness; W box: elicitation; wounding and pathogen responsiveness; GCC box: elicitation; wounding and pathogen responsiveness; motif IIb: abscisic acid responsive element; TC-rich repeats: cis-acting element involved in defense and stress responsiveness; TCA-element: cis-acting element involved in salicylic acid responsiveness; TGA-element:auxin-responsive element; HSE: cis-acting element involved in heat stress responsiveness; TGACG-motif: cis-acting regulatory element involved in the MeJA-responsiveness. The 1.5 kb sequences upstream of the initiation codon (ATG) of the *JHDM* genes can be estimated using the scale per 250 bp at the above

To further elucidate the possible regulation mechanisms of maize *JHDM* genes in the abiotic or biotic stress response, the promoter sequences were analyzed using the PlantCARE database to identify cis-regulatory elements in the promoter region. Twelve types of stress- and hormone-related cis-acting regulatory elements were detected in the promoters of maize *JHDM* genes: eight hormone-related elements (ERE, ABRE, GARE-motif, CGTCA-motif, motif IIb, TCA-element, TGA-element, TGACG-motif) and four stress-related elements (TC-rich repeats, W box, GCC box, HSE) (Fig. 5). All 19 *JHDM* genes contained 3–8 cis-elements related to stress or hormone response. The ABRE (abscisic acid responsiveness) were detected in 15 and the CGTCA-motif (MeJA-responsiveness) and TGACG-motif (MeJA-responsiveness) were detected in 13 maize *JHDM* genes. In addition, 11, 10, 9, 9 and 7 *JHDM* genes contained TGA-element (auxin-responsive element), TC-rich repeats (defense and stress responsiveness), TCA-element (salicylic acid responsiveness), W-box (wounding and pathogen responsiveness), and GARE-motif (gibberellin-responsive element), respectively. Therefore, these results demonstrated that expression of maize *JHDM* genes would be regulated by various environmental factors.

### Analysis of microarray expression profile of maize *JHDM* genes

**Fig. 6:**
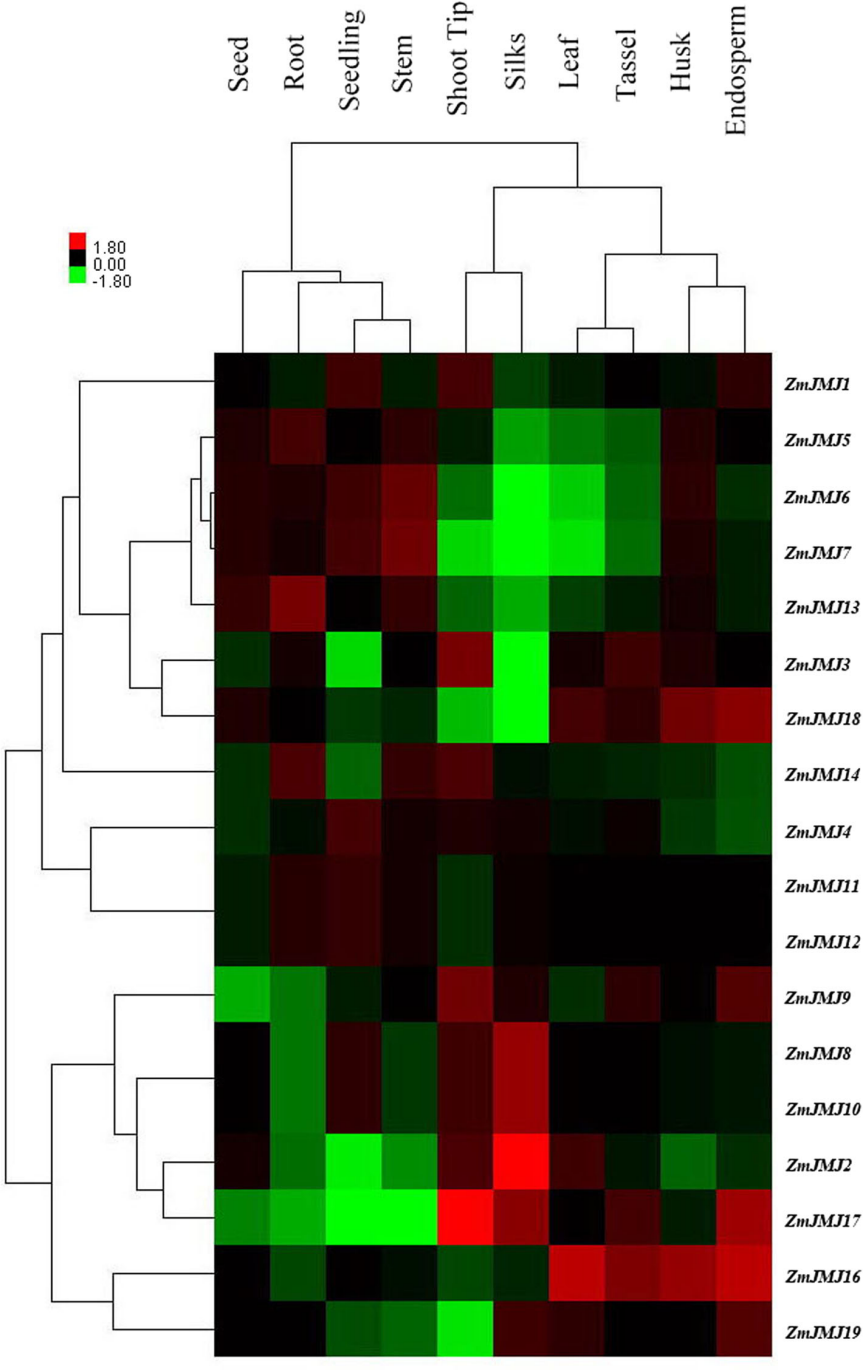
Hierarchical clustering of expression profiles of 19 *ZmJMJ* genes in 10 tissues. The color scale representing log2 signal values is shown on the left. Green represents a low level and red indicates a high level of transcript abundance (colour figue online). The different tissues and/or organs are noted on the top of each lane. Cluster dendrograms are shown on the left and above, respectively

To further investigate the possible functions of the *ZmJMJ*s in plant growth and development, a comprehensive expression analysis was firstly accomplished based on whole-genome coverage according to the microarray data released by Sekhon et al. (2011) [29], which provide us with valuable information for gene functional characterization. It can be seen from the heat map that 18 of all 19 *ZmJMJ* genes (except *ZmJMJ15*) have distinct expression levels in 10 tissues at different developmental stages as shown in Additional file 1: Table S1, which can be divided into two major groups (Fig. 6). The first group includes all the members of the JADID1 subfamily (*ZmJMJ5, ZmJMJ6, ZmJMJ7, ZmJMJ13* and *ZmJMJ14*), the JHDM3 subfamily (*ZmJMJ1, ZmJMJ3, ZmJMJ11* and *ZmJMJ18*), and the JHDM2 subfamily (*ZmJMJ4*), which exhibit relatively low expression levels in most of the tissues and/or organs, while the second group, containing all the members of the JADID1 subfamily (*ZmJMJ10* and *ZmJMJ16*), the JHDM3 subfamily (*ZmJMJ9, ZmJMJ12, ZmJMJ17* and *ZmJMJ19*), and the JHDM2 subfamily (*ZmJMJ2* and *ZmJMJ8*), show relatively high expression levels. In addition, some tissue/organ-specific genes were discovered according to the results shown in the heat map. Specifically, *ZmJMJ2* and *ZmJMJ17* were expressed at high levels in silk and shoot tips respectively, which was remarkably more than any other tissues (Additional file 5: Table S2). Moreover, to further confirm the expression patterns of 19 *JHDM* genes in maize, we also carried out a comprehensive expression profile analysis of the maize *JHDM* gene family using GENEVESTIGATOR database (https://genevestigator.com/gv/index.jsp) (Additional file 6: Figure S4). The results further demonstrated that the maize *JHDM* gene family displayed different expression patterns at diverse developmental stages of maize. Furthermore, to investigate stress-responsiveness of these *JHDM* genes to environmental stress, we also carried out a comprehensive expression profile analysis of the maize *JHDM* gene family by using GENEVESTIGATOR database (Additional file 7: Figure S5). The results further demonstrated that the maize *JHDM* gene family displayed different expression patterns under diverse environmental stress conditions, suggesting that these genes were responsive to stress treatments.

### Expression analysis of maize *JHDM* genes under heat stress treatment

**Fig. 7:**
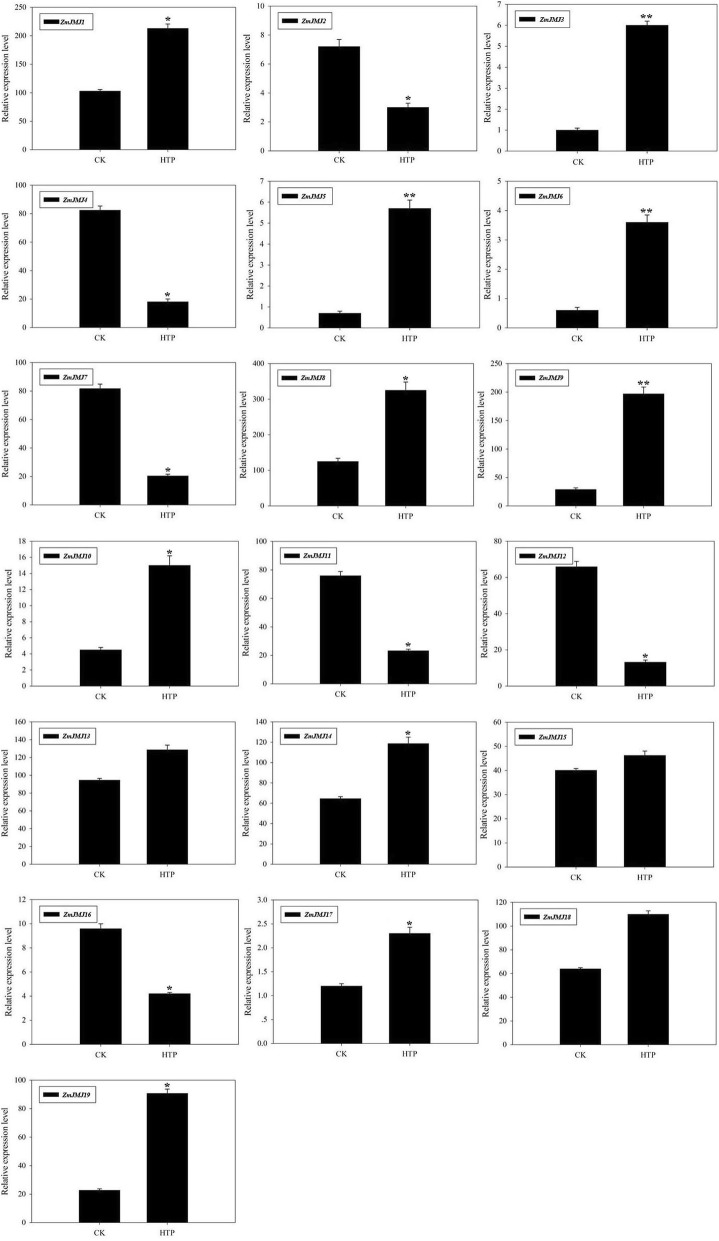
Relative gene expression of *ZmJMJs* analyzed by qRT-PCR response to heat stress treatment. QRT-PCR data was normalized using maize *Actin* gene and are shown relative to CK (normal condition). X-axes show different treatments (CK, normal condition and HTP, heat stress condition) and y-axes are scales of relative expression level. Bars represent standard deviations (SD) of three technical replicates. A significant difference is indicated by asterisks according to the *t* test (*P < 0.05, **P < 0.01)

To survey if these predicted genes were expressed in maize and to further confirm their stress-responsiveness to abiotic stress, the transcripts of the 19 genes were firstly detected in the leaves by heat treatment using RT-PCR (Additional file 8: Figure S6). The results revealed that all 19 *ZmJMJ* genes were responsive to heat stress treatment, suggesting the authenticity of these genes and their potential roles in heat stress response. Secondly, to further confirm their stress-responsiveness to abiotic stress, quantitative real-time PCR was accomplished for 19 *ZmJMJ* genes in the leaves of maize exposed to heat stress (Fig. 7). Three biological replications were performed in all reactions. The results revealed that these genes are differentially expressed in the leaves under either normal condition (CK) or heat stress condition (HTP). Interestingly, most of members of *ZmJMJ* gene family exhibited similar up-regulated expressional patterns under heat stress treatment. Among them, there were seven of the expressed genes (*ZmJMJ3, ZmJMJ5, ZmJMJ6, ZmJMJ8, ZmJMJ9, ZmJMJ10 and ZmJMJ19*), which had lower expression level in the leaves of maize under normal condition, however, they showed significantly up-regulated expression levels (> 2-fold) during heat stress treatment. What’s more, six expressed genes (*ZmJMJ2, ZmJMJ4, ZmJMJ7, ZmJMJ11, ZmJMJ12 and ZmJMJ16*) were greatly down-regulated (< 0.5-fold) during the heat stress treatment. These results demonstrated that these predicted genes of the *ZmJMJ* gene family were responsive to heat stress treatment, suggesting their potential roles in heat stress response. Additionally, to further demonstrate that these predicted genes of the *ZmJMJ* gene family were responsive to heat stress treatment, we also constructed the expression profile of 22 newly-identified heat shock protein genes (*ZmHSP70–1*~*ZmHSP70–22*) (unpublished), which may play a central role in the heat stress response (Additional file 9: Figure S7). The results revealed that most of the identified heat shock protein genes in maize exhibited significantly up-regulated expression levels.

## Discussion

Histone methylation plays an important role in epigenetic regulation of gene expression, which is determined by adjusting the homeostasis of histone methylation through histone methyltransferase and demethylase. JmjC domain-containing proteins represent a large family of histone demethylases in both animals and plants and play essential roles in histone modification, which is a significant part of epigenetics [7, 30]. In the present study, a comprehensive set of 19 no redundant *ZmJMJ* genes were identified and characterized from the latest version of the maize (B73) genome database, including their phylogenetic relationships, gene structure, domain architecture, chromosome location, duplication patterns, and expression profiles.

Initially, phylogenetic analysis provided insights into the evolution of gene family members and gene multiplicity in maize. In our investigation of JHDM proteins, we found that maize *JHDM* genes were mainly categorized into three distinct subclasses, which is also present in *Arabidopsis* and rice. However, in the JHDM6 or JmjC-only subfamily, no any *JHDM* gene was found in maize, indicating that the evolution of the *JHDM* genes in these two groups may occur after divergence of monocots and dicots. Moreover, although the maize genome size (2300Mbp) is larger than that of *Arabidopsis* (125Mbp) and rice (389Mbp), only 19 *JHDM* genes were identified and characterized in maize. The number of *ZmJMJ* genes is similar to previous studies in *Arabidopsis* (21) and rice (20). This phenomenon may result from less gene duplications in maize genome or experiencing a large gene loss during maize genome duplication. This further demonstrates that the JHDM proteins are highly conserved in evolution. Moreover, the structure features of ZmJMJ proteins were revealed preliminarily by analyzing the structures of ZmJMJ proteins. All of the ZmJMJ proteins are mainly sorted into three different subclasses and each contains the conserved JmjC domain of ZmJMJ protein. Remarkably, the majority of members of the JARID1 subfamily share FYRN and FYRC domains, which may possess chromatin-binding activity [11] or contribute to JmjC domain function by collaborating with other proteins. For instance, the functional specificity of AtJMJ14 in flowering time control has been demonstrated to be on account of the specificity of its interaction with transcription factors through the FYRC domain [31]. Moreover, the ARID domain (AT-rich interaction domain) contained in ZmJMJ10 has been revealed to be implicated in sequence-specific DNA binding in *Drosophila melanogaster* [32].

Furthermore, to further investigate the possible functions of maize *JHDM* genes in growth and development and stress-responsiveness of these *JHDM* genes to environmental stress, several distinct kinds of digital expression pattern analyses were conducted by applying different databases. Firstly, a comprehensive expression analysis was accomplished based on whole-genome coverage according to the microarray data released by Sekhon et al. (2011) [29]. As shown from the heat map in Fig. 6, the maize *JHDM* genes showed apparent differential expression patterns. Some genes such as *ZmJMJ1*, *ZmJMJ11* and *ZmJMJ12* appear to be invariable or lowly expressed among all tissues. By contrast, *ZmJMJ2*, *ZmJMJ16* and *ZmJMJ17* exhibited remarkably different expression levels in different tissues. For instance, the expression of *ZmJMJ2* in silk and *ZmJMJ17* in shoot tips was remarkably more than any other tissues. Furthermore, the expression level of *ZmJMJ*s was similar between seedling and stems, leaves and tassels or husks and endosperms. However, in the same tissues of maize, different *ZmJMJ*s exhibited huge diverse expression profiles. For example, in silks and shoot tips, some genes such as *ZmJMJ2* and *ZmJMJ17* showed a high expression level, whereas other genes like *ZmJMJ7* and *ZmJMJ18* exhibited a lower expression level. Taken together, most of *ZmJMJ*s exhibited apparent differential expression patterns. Interestingly, some genes with close relationship, such as *ZmJMJ8/ ZmJMJ10* and *ZmJMJ11/ZmJMJ12*, have similar expression patterns in different tissues, suggesting functional redundancy among these genes. Secondly, a comprehensive expression profile analysis of the maize *JHDM* gene family by using GENEVESTIGATOR database (Additional file 6: Figure S4) was further performed to demonstrate that the maize *JHDM* gene family displayed different expression patterns at diverse developmental stages of maize. Additionally, to further confirm stress-responsiveness of *JHDM* genes to environmental stress, we carried out a comprehensive expression profile analysis of the maize *JHDM* gene family using GENEVESTIGATOR database (Additional file 7: Figure S5), indicating that the maize *JHDM* gene family displayed different expression patterns under diverse environmental stress conditions, suggesting that these genes were responsive to stress treatments.

Eventually, to confirm these gene models and their stress-responsiveness to abiotic stress, the transcripts of the 19 genes were firstly detected in the leaves by heat treatment using RT-PCR (Additional file 8: Figure S6). Moreover, the expression analysis of quantitative RT-PCR (Fig. 7) also showed that maize *JHDM* genes exhibit different expression levels by heat stress. Thus, the results of semi-quantitative PCR and quantitative RT-PCR revealed the authenticity of these genes and their potential roles in heat stress response. In this study, we detected the majority of members from the JARID1 subfamily and the JHDM3 subfamily with significantly higher expression, when subjected to heat stress, indicating that these two subfamilies was closely correlated with maize heat stress response. Moreover, seven genes (*ZmJMJ3, ZmJMJ5, ZmJMJ6, ZmJMJ8, ZmJMJ9, ZmJMJ10 and ZmJMJ19*) of the *ZmJMJ* gene family were remarkably up-regulated under heat stress condition, which suggested specific roles for these genes in maize during heat stress. It is note-worthy that two duplicated pairs (*ZmJMJ5/ZmJMJ6*, *ZmJMJ3/ZmjMJ17*) in this study exhibited similar expression patterns under heat stress condition. It implied that the expression patterns among the surviving duplicated pairs are relatively conserved, suggesting little functional differentiation has occurred following the WGD/segmental events. Furthermore, heat shock proteins usually play a central role in the heat stress response and the accumulation of heat shock proteins might be a useful indicator for the heat stress treatment. Therefore, to further demonstrate that these predicted genes of the *ZmJMJ* gene family were responsive to heat stress treatment, a comprehensive expression profile analysis of 22 newly-identified heat shock protein genes (*ZmHSP70–1*~*ZmHSP70–22*) was detected in the leaves by heat treatment using RT-PCR (Additional file 9: Figure S7), indicating that these predicated genes may share potential roles in heat stress response. Above all, the analysis of these differentially expressed genes provides some novel insights into plants response to heat stress. However, the functions of targets genes still require further investigation.

## Conclusion

In our study, a total of 19 *JHDM* genes were identified in maize, distributing on nine chromosomes at different densities. These JHDM proteins were mainly categorized into three subfamilies based on the similarities of the amino acid sequences. The exon/intron composition, conserved domain, and amino acid residues were considerably conserved among the members of the same group or subgroup. The close phylogenetic relationship among ZmJMJs, OsJMJs and AtJMJs in the same subgroup provided insights into their putative functions. A comprehensive expression profile of all members of *JHDM* gene family in maize suggest that many maize *JHDM* genes play functional developmental roles in multiple tissues. Furthermore, the expression profiles by RT-PCR and quantitative real-time PCR revealed that the majority of identified *ZmJMJs* most likely are expressed in maize and these genes are induced by heat stress with differential induction levels in leaves. Taken together, all of these results provided valuable clues in future efforts to identify specific gene functions of this gene family and gene diversity among different genotype of maize and other plants in monocots.

## Methods

### Identification of the members of *JHDM* gene family in maize

To identify JmjC domain-containing proteins in maize, the maize genomic database (http://www.maizesequence.org/index.html) was searched using Basic Local Alignment Search Tool algorithms (BLASTP) (*P* value = 0.001), with the published 21 *Arabidopsis* and 20 rice JmjC domain-containing protein sequences and their JmjC domain used as initial query sequences, respectively. Redundant sequences were then removed manually, and the Hidden Markov Model (HMM) profile of the JmjC domain (PF02373, SM00558) was obtained from Pfam database [33] (http://pfam.xfam.org/) and SMART database [34] (http://smart.embl-heidelberg.de/) to determine each candidate protein as a member of the *ZmJMJ* gene family [35]. The newly identified genes were designated on the basis of their phylogenetic relatedness to other members of the same gene family in *Arabidopsis* and rice. Maize *JHDM* gene basic information including the number of amino acids, ORF lengths and chromosome locations was obtained from the B73 maize sequence database. Physicochemical parameters including the molecular weight (kDa) and isoelectric point (pI) of each gene product were calculated using compute pI/Mw tool from ExPASy (http://www.expasy.org/tools/) and parameter was set to average [36]. The exon and intron structures of individual maize *JHDM* genes were determined using GSDS (Gene structure display server; http://gsds.cbi.pku.edu.cn/) via alignment of the cDNAs with their corresponding genomic DNA sequences. The conserved domains in the JmjC domain-containing proteins encoded by *ZmJMJ* genes were investigated by using Pfam (http://pfam.xfam.org/) and SMART (http://smart.embl-heidelberg.de/) with default parameters. Moreover, the conserved amino acid residues for interaction with co-factors were also deduced by using Clustal-W software using default parameters [37].

### Phylogenetic analysis of maize JHDM proteins

Multiple sequence alignment of all predicted maize JmjC domain-containing protein sequences and the orthologs from *Arabidopsis* and rice was performed with Clustal-W software using default parameters. All protein sequences were downloaded from National Center for Biotechnology Information (NCBI). Then, based on this alignment, the analysis of phylogenetic relationship from three different species was conducted using the MEGA 6.0 program [38] with the neighbor-joining method and 1000 bootstrap replicates were performed.

### Chromosomal localization, synteny analysis and gene duplication of maize *JHDM* genes

The chromosomal location image of *ZmJMJ* genes was generated by MapInspect (https://mapinspect.software.informer.com/) according to their starting positions in maize chromosomes. To compare the genomic context of maize *JHDM* genes with that in other grass species, information on their patterns of microsynteny was retrieved from the database Phytozome v12.1 (www.phytozome.net). Orthologs of maize *JHDM* genes in other grasses were investigated in the CoGe database [29]. Duplication analysis of maize *JHDM* genes was carried out in the Synteny Mapping and Analysis Program (SyMAP) v4.2 [27] and WGMapping program in the PLAZA 4.0 platform [28]. To identify tandem and segmental duplications, two genes in the same species located in the same clade of the phylogenetic tree were defined as paralogs according to described previously [39, 40]. Two *JHDM*s placed on the synteny blocks in maize genome were designated as segmental duplicated paralogs, and two *JHDM*s separated by five or fewer gene loci were regarded as tandem duplicated paralogs as described [41, 42].

### Prediction of cis-acting elements in maize *JHDM* genes

Stress response and hormone-related cis-acting elements in the promoter sequences of the *JHDM* genes were surveyed using the PlantCARE database (http://bioinformatics.psb.ugent.be/webtools/plantcare/html) [43] by means of subjected the 1.5 kb sequences upstream of the initiation codon (ATG) of each *JHDM* genes.

### Plant materials and stress treatment

Maize (*Zea mays* L. inbred line B73) plants were grown in a growth chamber at 28 °C with a photoperiod of 15-h light and 9-h dark and a relative humidity of 60%. Three-week-old seedlings (five-leaf stage) of maize were used to examine the expression patterns of *ZmJMJ* genes under heat stress. For heat treatment, seedling were incubated in an incubator at 42 °C in the dark for 8 h. Seedlings maintained with enough water at 28 °C in the dark were used as controls, and seedling leaves were sampled after heat treatment, frozen in liquid nitrogen, and stored at − 80 °C.

### Expression profile analysis of maize *JHDM* genes

To determine the expression patterns of the *JHDM* genes in maize tissues, a comprehensive expression analysis was initially performed based on whole-genome coverage according to the microarray data released by Sekhon et al. (2011) [29], which was publically available at MaizeGDB (http://www.maizegdb.org). Microarray analysis was accomplished to identify expression patterns in representative tissues, including seed, root, seedling, stem, shoot tip, silks, leaf, tassel, husk and endosperm. By using Cluster 3.0 (http://bonsai.hgc.jp/~mdehoon/software/cluster/software.htm) and Treeview software (http://jtreeview.sourceforge.net/), we carried out the hierarchical clustering analysis based on the Pearson coefficients with average linkage and analyzed the corresponding gene expression patterns in multiple different tissues or organs, respectively. Moreover, a comprehensive expression profile analysis of the maize *JHDM* gene family using GENEVESTIGATOR database (https://genevestigator.com/gv/index.jsp) was also conducted to further confirm the expression patterns of 19 *JHDM* genes in maize. Furthermore, a semi-quantitative expression profile was firstly analyzed in the leaves by heat stress treatment and a quantitative real-time PCR analysis of expression profile was further performed in the leaves by heat stress treatment to reveal the impact of external environmental factor on expression of *ZmJMJ* genes in maize. Total RNA was isolated from the collected samples using Trizol reagent (Invitrogen, USA), followed by DNase I treatment to remove any genomic DNA contamination. The first-strand cDNA was synthesized from 1 μg of total RNA using QuantiTect Rev.Transcription Kit (Qiagen, Germany). Gene-specific RT-PCR and qRT-PCR primers were designed according to their CDSs (Additional file 10: Table S3 and Additional file 11: Table S4) and then synthesized commercially (Generay), respectively. Quantitative RT-PCR was carried out using an ABI PRISM 7300 real-time PCR system (Applied Biosystems, USA). The qRT-PCR machine was set with 40 cycles and an annealing temperature of 60 °C. At least three biological replicates were performed per cDNA sample. The maize *Actin* gene was used as internal control for normalization. The relative mRNA level for each gene was calculated as 2^-ΔΔCT^ method to calculate the fold change in the expression level of the relevant genes. Statistical analyses were performed using SDS software version 1.3.1 (Applied Biosystems, USA). Subsequently, significant difference analysis was performed by Student’s *t* test (http://www.physics.csbsju.edu/stats/t-test_bulk_form.html), at significance level of *P* < 0.05.

## Additional files

Additional file 1: **Table S1.** Basic information of *JHDM* genes identified in maize. (DOC 48 kb)

Additional file 2: **Figure S1.** KDM5/JARID1 group proteins contain potential H3K4 demethylases in *Arabidopsis*, rice and maize. (JPG 375 kb)

Additional file 3: **Figure S2.** KDM4/JHDM3 group proteins are potential active histone demethylases in *Arabidopsis*, rice and maize. (JPG 396 kb)

Additional file 4: **Figure S3.** KDM3/JHDM2 group proteins contain potential H3K9 demethylases in *Arabidopsis*, rice and maize. (JPG 668 kb)

Additional file 5: **Table S2.** KDM3/JHDM2 group proteins contain potential H3K9 demethylases in *Arabidopsis*, rice and maize. The original data of expression profiles of *ZmJMJ1-19* genes (except *ZmJMJ15*) in 10 tissues. (DOC 76 kb)

Additional file 6: **Figure S4.** Hierarchical clustering of developmental expression patterns of maize *ZmJMJ* genes by Genevestigator database. (JPG 89 kb)

Additional file 7: **Figure S5.** Hierarchical clustering of expression patterns of maize *ZmJMJ* genes under diverse environmental stress conditions by Genevestigator database. (JPG 1.79 kb)

Additional file 8: **Figure S6.** Semi-quantitative RT-PCR analysis of *ZmJMJ* genes under heat stress treatment. (JPG 57.9 kb)

Additional file 9: **Figure S7.** Semi-quantitative RT-PCR analysis of *ZmHsp70* genes under heat stress treatment. (JPG 71 kb)

Additional file 10: **Table S3.** RT-PCR primers used in this study. (DOC 43 kb)

Additional file 11: **Table S4.** QRT-PCR primers used in this study. (DOC 42 kb)

## Abbreviations

ARID: AT-rich interaction domain
BR: Brassinosteroid
ELF6: Early flowering 6
FAD: Flavin adenine dinucleotide
FLC: Flowering locus C
FYRC: FY-rich domain C-terminal
FYRN: FY-rich domain N-terminal
IBM1: Increase in bonsai methylation 1
JARID: Jumonji, AT-rich interactive domain
JHDM: JmjC domain-containing histone demethylase
JMJ: JmjC domain protein
JmjC: Jumonji C
KDM: lysine demethylase
LSD: lysine-specific demethylase
LSDs: lysine-specific demethylases
PHD: plant home box domain
PRMT: Protein arginine methyltransferase
qRT-PCR: quantitative real-time PCR
REF6: Relative of early flowering 6
SET: Su (var) 3–9, Enhancer-of-zeste and Trithorax
а-KG: а-ketoglutarate
