# Supplementary material for: Genome-wide identification, classification and expression analysis of the JmjC domain-containing histone demethylase gene family in maize
Source: BMC Genomics. 2019 Apr 1;20:256. doi: 10.1186/s12864-019-5633-1 (PMC6444447; doi:10.1186/s12864-019-5633-1)
Supplement: Supplementary file 1 — Table S1. Basic information of JHDM genes identified in maize. (DOC 48 kb) [file 12864_2019_5633_MOESM1_ESM.doc]

**Table S1 Basic information of *JHDM*** genes identified in maize

| **Serial**  **NO.** | **Gene Name** | **Accession Number**  **Ensembl transcript** |  | **Genome Location Coordinates (5'-3')** | **ORF**  **Length**  **(bp)** | **Length**  **(a.a.)** | **Protein**  **Mol.Wt.**  **(Da)** | **PI** | **Chr.** | **Type** |
| --- | --- | --- | --- | --- | --- | --- | --- | --- | --- | --- |
| **1**  **2**  **3**  **4**  **5**  **6**  **7**  **8**  **9**  **10**  **11**  **12**  **13**  **14**  **15**  **16**  **17**  **18**  **19** | *ZmJMJ1*  *ZmJMJ2*  *ZmJMJ3*  *ZmJMJ4*  *ZmJMJ5*  *ZmJMJ6*  *ZmJMJ7*  *ZmJMJ8*  *ZmJMJ9*  *ZmJMJ10*  *ZmJMJ11*  *ZmJMJ12*  *ZmJMJ13*  *ZmJMJ14*  *ZmJMJ15*  *ZmJMJ16*  *ZmJMJ17*  *ZmJMJ18*  *ZmJMJ19* | GRMZM2G057466_P01  GRMZM2G417089_P01  GRMZM2G321810_P01  GRMZM2G383210_P01  GRMZM2G107109_P02  GRMZM2G140524_P02  GRMZM2G027075_P02  GRMZM2G054162_P01  GRMZM2G180086_P01  GRMZM2G466292_P01  GRMZM2G060919_P01  GRMZM2G060919_P02  GRMZM2G156910_P01  GRMZM2G156910_P02  GRMZM2G044301_P01  GRMZM2G108589_P01  GRMZM2G339379_P01  AC149475.2_FGP005  GRMZM2G431157_P01 |  | 103615815..103621968248509167..248516128  165449191..165455337169209879..169229732  172257227..172265943172309403..172318450  172430278..172438802240328297..240340399  44629098..44635216  65425225..65434544  199221816..199229761  199223522..199227476  116474452..116480643116474465..116480643  134137146..134143314  1516447..1519778  163447221..163451966  152524931..152535598  19846289..19854107 | 1500  3153  3240  3477  3828  3699  2358  3000  2709  1584  1803  2043  3564  3708  2355  1764  2610  4569  3429 | 499  1050  1079  1148  1257  1232  785  999  902  527  600  680  1187  1235  784  587  870  1522  1142 | 56430.79  119511.63  119415.97  127784.49  139665.24  136581.68  88258.34  111903.46  101338.68  59810.97  68027.90  76922.81  133048.76  138597.01  87770.39  66712.51  94612.68  167759.67  127258.92 | 3.69  8.82  9.25  7.77  8.03  7.66  7.23  7.59  8.93  9.71  6.45  6.17  6.94  6.38  5.47  6.72  6.73  5.98  8.59 | 1  1  3  4  4  4  4  4  5  5  5  5  6  6  6  7  8  9  10 | II  III  II  III  I  I  I  III  II  I  II  II  I  I  I  I  II  II  II |

**Note:** the information of maize *JHDM* gene family, including chromosomal location and ORF length, were retrieved from the B73 maize sequencing database (http://www.maize sequence.org/index.html). I, the *JARID1* subfamily; II, the *JHDM3* subfamily; III, the *JHDM2* subfamily.
