# Supplementary figures and images for: Genome-wide identification, classification and expression analysis of the JmjC domain-containing histone demethylase gene family in maize
Source: BMC Genomics. 2019 Apr 1;20:256. doi: 10.1186/s12864-019-5633-1 (PMC6444447; doi:10.1186/s12864-019-5633-1)

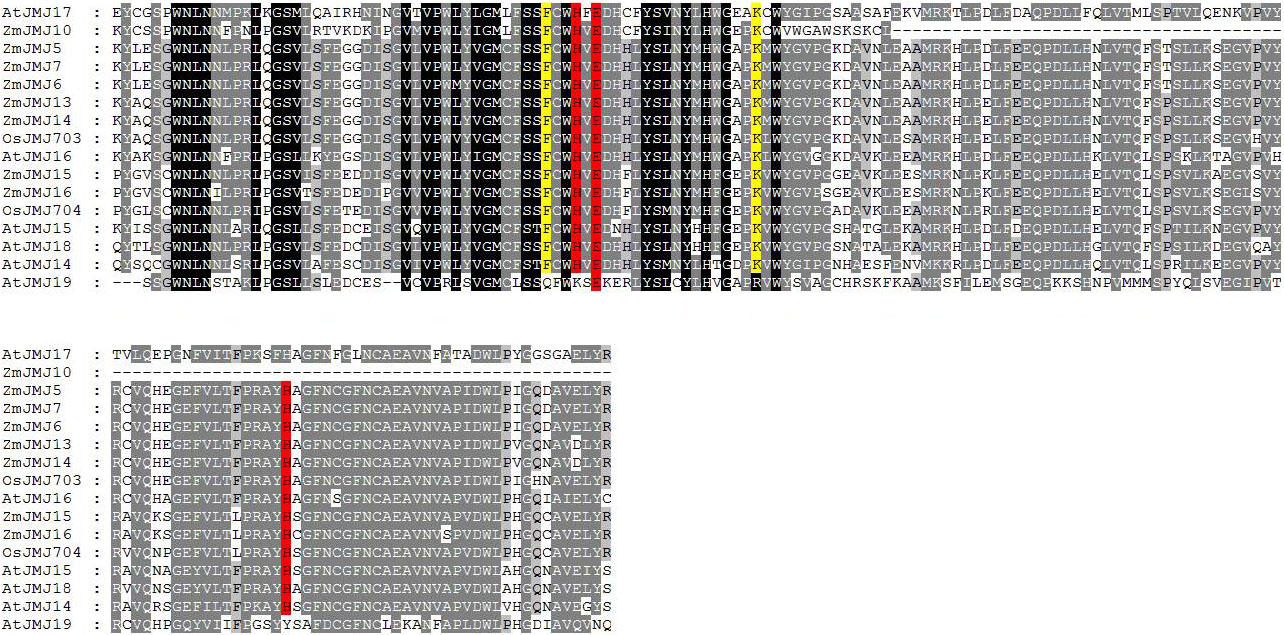

Supplement: Supplementary file 2 — Figure S1. KDM5/JARID1 group proteins contain potential H3K4 demethylases in Arabidopsis, rice and maize. (JPG 375 kb) [file 12864_2019_5633_MOESM2_ESM.jpg]

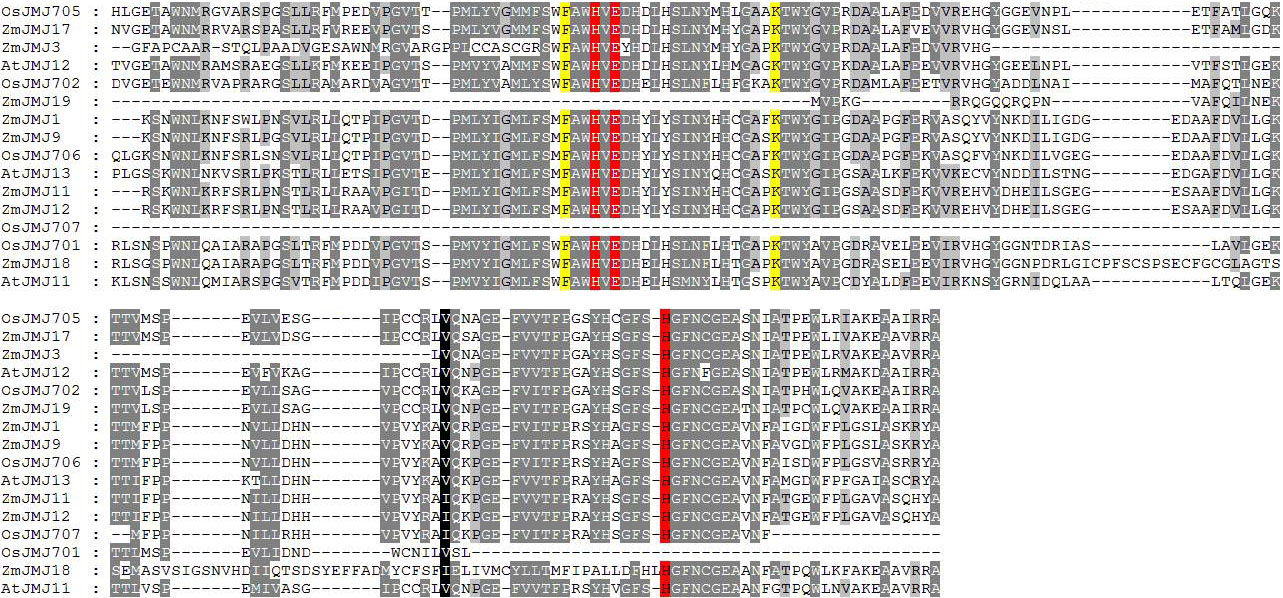

Supplement: Supplementary file 3 — Figure S2. KDM4/JHDM3 group proteins are potential active histone demethylases in Arabidopsis, rice and maize. (JPG 396 kb) [file 12864_2019_5633_MOESM3_ESM.jpg]

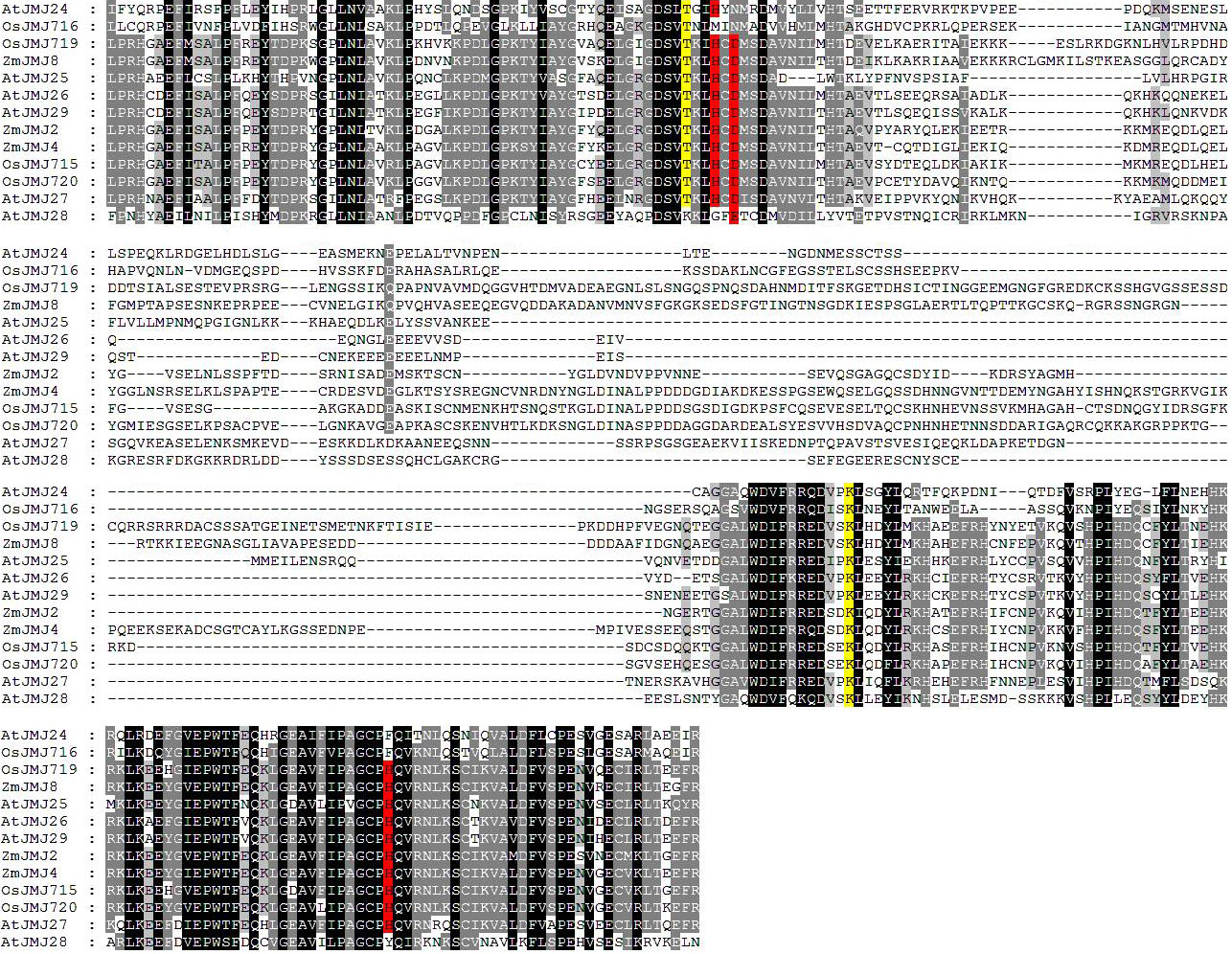

Supplement: Supplementary file 4 — Figure S3. KDM3/JHDM2 group proteins contain potential H3K9 demethylases in Arabidopsis, rice and maize. (JPG 668 kb) [file 12864_2019_5633_MOESM4_ESM.jpg]

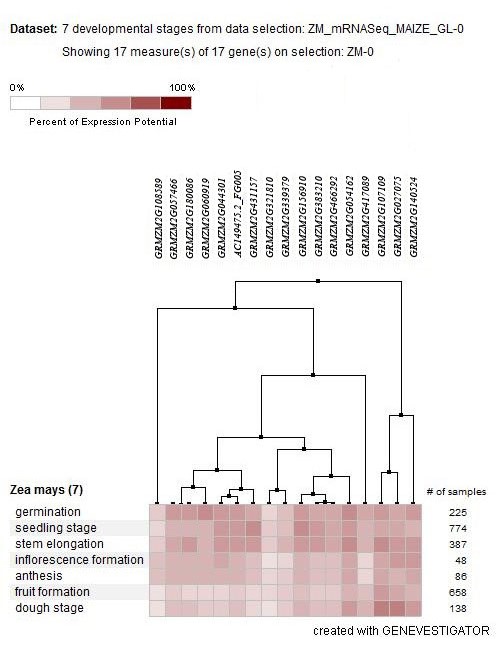

Supplement: Supplementary file 6 — Figure S4. Hierarchical clustering of developmental expression patterns of maize ZmJMJ genes by Genevestigator database. (JPG 89 kb) [file 12864_2019_5633_MOESM6_ESM.jpg]

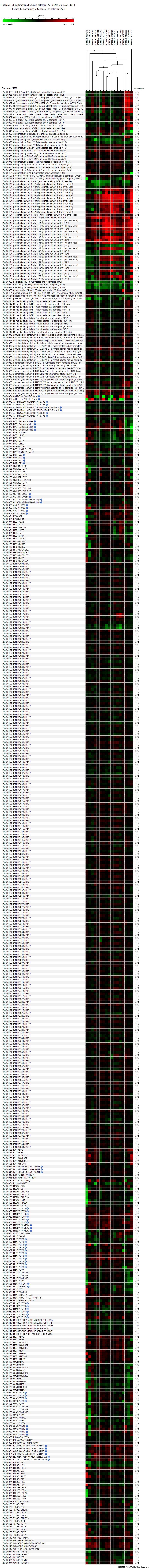

Supplement: Supplementary file 7 — Figure S5. Hierarchical clustering of expression patterns of maize ZmJMJ genes under diverse environmental stress conditions by Genevestigator database. (JPG 1.79 kb) [file 12864_2019_5633_MOESM7_ESM.jpg]

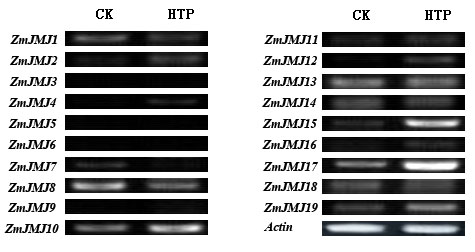

Supplement: Supplementary file 8 — Figure S6. Semi-quantitative RT-PCR analysis of ZmJMJ genes under heat stress treatment. (JPG 57.9 kb) [file 12864_2019_5633_MOESM8_ESM.jpg]

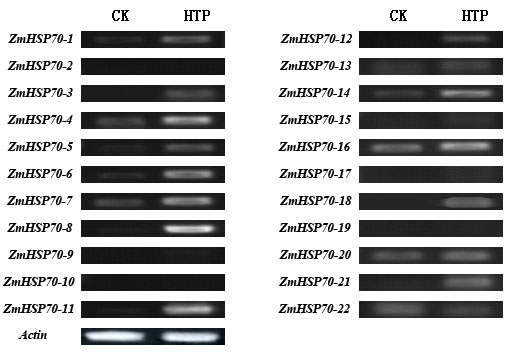

Supplement: Supplementary file 9 — Figure S7. Semi-quantitative RT-PCR analysis of ZmHsp70 genes under heat stress treatment. (JPG 71 kb) [file 12864_2019_5633_MOESM9_ESM.jpg]
