# Supplementary material for: Genome-wide identification, classification and expression analysis of the JmjC domain-containing histone demethylase gene family in maize
Source: BMC Genomics. 2019 Apr 1;20:256. doi: 10.1186/s12864-019-5633-1 (PMC6444447; doi:10.1186/s12864-019-5633-1)
Supplement: Supplementary file 5 — Table S2. KDM3/JHDM2 group proteins contain potential H3K9 demethylases in Arabidopsis, rice and maize. The original data of expression profiles of ZmJMJ1-19 genes (except ZmJMJ15) in 10 tissues. (DOC 76 kb) [file 12864_2019_5633_MOESM5_ESM.doc]

**Table S2 The original data of expression profiles of *ZmJMJ1-19* genes (except *ZmJMJ15*)** in 10 tissues

| **Gene** | **Seed** | **Root** | **Seedling** | **Stem** | **Shoot Tip** | **Silks** | **Leaf** | **Tassel** | **Husk** | **Endosperm** | **Average** |  |
| --- | --- | --- | --- | --- | --- | --- | --- | --- | --- | --- | --- | --- |
| *ZmJMJ5* | 10.04 | 10.45 | 10.39 | 9.3 | 9.56 | 9.81 | 10.37 | 10.16 | 10.35 | 9.34 | **9.977** |  |
| *ZmJMJ6* | 10.01 | 10.76 | 10.02 | 9.27 | 10.06 | 10.78 | 10.3 | 10.06 | 10.33 | 7.9 | **9.949** |  |
| *ZmJMJ7* | 8.32 | 9.75 | 10.07 | 8.14 | 9.04 | 9.86 | 10.25 | 10.19 | 10.43 | 7.89 | **9.394** |  |
| *ZmJMJ13* | 9.69 | 7.79 | 7.61 | 8.53 | 9.18 | 8.25 | 9.08 | 10.04 | 8.66 | 9.36 | **8.819** |  |
| *ZmJMJ14* | 9.14 | 8.88 | 8.21 | 8.34 | 8.34 | 7.46 | 8.51 | 8.63 | 8.78 | 8.75 | **8.504** |  |
| *ZmJMJ1* | 10.88 | 10.15 | 10.34 | 10.11 | 10.41 | 10.42 | 10.5 | 11.28 | 10.04 | 10.24 | **10.437** | **Low** |
| *ZmJMJ3* | 10.65 | 9.68 | 9.41 | 9.86 | 10.22 | 7.82 | 10.22 | 10.4 | 9.69 | 7.78 | **9.573** |  |
| *ZmJMJ11* | 9.85 | 10.21 | 9.95 | 10.14 | 10.17 | 10.1 | 10.42 | 10.78 | 10.13 | 10.52 | **10.227** |  |
| *ZmJMJ18* | 8.11 | 9.24 | 9.59 | 9.89 | 9.77 | 8.61 | 10.45 | 11 | 8.98 | 5.68 | **9.132** |  |
| *ZmJMJ4* | 8.55 | 11.42 | 11.39 | 11.59 | 11.83 | 11.83 | 7.53 | 8.25 | 6.84 | 10.38 | **9.961** |  |
|  |  |  |  |  |  |  |  |  |  |  |  |  |
| *ZmJMJ10* | 13.97 | 12.47 | 13.54 | 13.47 | 13.48 | 13.4 | 13.64 | 13.94 | 12.98 | 14.83 | **13.572** |  |
| *ZmJMJ16* | 9.72 | 9.41 | 10.12 | 12.44 | 12.06 | 9.7 | 11.47 | 13.07 | 9.91 | 12.18 | **11.008** |  |
| *ZmJMJ9* | 11.05 | 12.33 | 12.06 | 11.22 | 11.52 | 11.28 | 12.1 | 12.12 | 11.92 | 10.79 | **11.639** |  |
| *ZmJMJ12* | 10.85 | 11.21 | 10.95 | 11.14 | 11.17 | 11.1 | 11.42 | 11.78 | 11.13 | 11.52 | **11.227** | **high** |
| *ZmJMJ17* | 10.18 | 10.32 | 10.82 | 11.77 | 22.28 | 18.48 | 11.79 | 13.49 | 11.91 | 13.03 | **13.407** |  |
| *ZmJMJ19* | 9.33 | 11.62 | 10.88 | 11.17 | 10.92 | 9.92 | 11.1 | 13.08 | 10.03 | 12.63 | **11.068** |  |
| *ZmJMJ2* | 13.97 | 10.02 | 9.11 | 9.73 | 10.88 | 17.91 | 13.61 | 13.76 | 13.74 | 14.19 | **12.692** |  |
| *ZmJMJ8* | 13.97 | 12.47 | 13.54 | 13.47 | 13.48 | 13.4 | 13.64 | 13.94 | 12.98 | 14.83 | **13.572** |  |
