# Supplementary material for: Genome-wide identification, classification and expression analysis of the JmjC domain-containing histone demethylase gene family in maize
Source: BMC Genomics. 2019 Apr 1;20:256. doi: 10.1186/s12864-019-5633-1 (PMC6444447; doi:10.1186/s12864-019-5633-1)
Supplement: Supplementary file 10 — Table S3. RT-PCR primers used in this study. (DOC 43 kb) [file 12864_2019_5633_MOESM10_ESM.doc]

**Table S3 RT-PCR primers used in this study.**

| Primer | Sequence (5'to3') | Amplicon size (bp) |
| --- | --- | --- |
| ZmJMJ1-F  ZmJMJ1-R  ZmJMJ2-F  ZmJMJ2-R  ZmJMJ3-F  ZmJMJ3-R  ZmJMJ4-F  ZmJMJ4-R  ZmJMJ5-F  ZmJMJ5-R  ZmJMJ6-F  ZmJMJ6-R  ZmJMJ7-F  ZmJMJ7-R  ZmJMJ8-F  ZmJMJ8-R  ZmJMJ9-F  ZmJMJ9-R  ZmJMJ10-F  ZmJMJ10-R  ZmJMJ11-F  ZmJMJ11-R  ZmJMJ12-F  ZmJMJ12-R  ZmJMJ13-F  ZmJMJ13-R  ZmJMJ14-F  ZmJMJ14-R  ZmJMJ15-F  ZmJMJ15-R  ZmJMJ16-F  ZmJMJ16-R  ZmJMJ17-F  ZmJMJ17-R  ZmJMJ18-F  ZmJMJ18-R  ZmJMJ19-F  ZmJMJ19-R  Actin-F  Actin-R | ATTTCACCTGAGGCTGCTA  AAGGCACTCCCATCAACG  GACAGGAGCAGATGGAGG  CTTTGGTGGGCAAGGTAT  CAAATAGTGATTGGGCTGTG  TTTCTCCCTCTTGCTCGT  CACCGACGACCAGACCTT  TTTGCTCCTTGCTGCCTC  TCCCAAAGTCTTCTCCCG  TTTCCTCATTGCTGCCTC  TCCCAAAGTCTTCTCCCG  TTTCCTCATTGCTGCCTC  CTTCAGGGACCCATAGTTG  CTTCCTCCGCTTCTTCAT  GAACATTGACTCAGCCTAC  GATAAATACTGCCTCACCTA  TTGTGATGTTGATGGGAGTG  TGAAGCCTGCGTGGTAAG  CCATCGGTTTCTCGTTCA  AATGCCAGCCTTTATCACA  GGGATGCTCTTTAGTATGT  AGGTTTCTGTATGGCTCT  GAATGGACCAAGGATGAC  AGAAGACGCAGCGTAGAG  TGAAGTAGCAGACAAGGGTG  GCTGGTGGAACAATACGG  TGAAGTAGCAGACAAGGGTG  GCTGGTGGAACAATACGG  CCTCTTGGACCATTGTTT  CTGGCAGTTTAGCCTTCT  ATTTGCCGTATCATCCCA  TTCCTGCTTGAACCCATT  AGGACCAACTTCCCATAGC  AACAGGGTAACGAACGACT  CAGCGGCACAAGAAACGG  ACATTCAGACGGCGAGCA  AACACCCGACATCATCAA  TTTCTTACCCATCTTTACC  ATGGCTGACGGTGAG  TTAGAAGCACTTCCG | 353  437  413  439  293  293  500  413  479  388  279  302  258  258  337  309  461  384  309  455 |
