# Supplementary material for: Genome-wide identification, classification and expression analysis of the JmjC domain-containing histone demethylase gene family in maize
Source: BMC Genomics. 2019 Apr 1;20:256. doi: 10.1186/s12864-019-5633-1 (PMC6444447; doi:10.1186/s12864-019-5633-1)
Supplement: Supplementary file 11 — Table S4. QRT-PCR primers used in this study. (DOC 42 kb) [file 12864_2019_5633_MOESM11_ESM.doc]

**Table S4 qRT-PCR primers used in this study.**

| Primer | Sequence (5'to3') | Amplicon size (bp) |
| --- | --- | --- |
| ZmJMJ1-F  ZmJMJ1-R  ZmJMJ2-F  ZmJMJ2-R  ZmJMJ3-F  ZmJMJ3-R  ZmJMJ4-F  ZmJMJ4-R  ZmJMJ5-F  ZmJMJ5-R  ZmJMJ6-F  ZmJMJ6-R  ZmJMJ7-F  ZmJMJ7-R  ZmJMJ8-F  ZmJMJ8-R  ZmJMJ9-F  ZmJMJ9-R  ZmJMJ10-F  ZmJMJ10-R  ZmJMJ11-F  ZmJMJ11-R  ZmJMJ12-F  ZmJMJ12-R  ZmJMJ13-F  ZmJMJ13-R  ZmJMJ14-F  ZmJMJ14-R  ZmJMJ15-F  ZmJMJ15-R  ZmJMJ16-F  ZmJMJ16-R  ZmJMJ17-F  ZmJMJ17-R  ZmJMJ18-F  ZmJMJ18-R  ZmJMJ19-F  ZmJMJ19-R  Actin-F  Actin-R | ATGTCCAACCTTGAGTGGATAG  TACCATATTTAGCAGCCTCAGG  CGAGAGACTGCAAATACGAAAG  TTTTGTCCAGTGCATCTGAAAG  TTGAAGGAAAAACGAGCAAGAG  GTTTGAGCTTCTTGGTTTTTGC  TCTCGGAATCGTGGTTAATTGA  GATGATATTCATGTCACTCGCC  AGAAGACCAGGTGGAACATATG  GACAATCCTCATCACGAGTTGA  TTTCTGAATCTGTTCGTCTCGT  CTAGCTCTCTTACGTTTCGACT  GTGACGAATGCCTTCAAAGAAA  GAATCGATGTACAACGTTAGCC  GCAAACGTGTGTATGAAACTCT  GTGCAACAAGTGAGTTTACAGT  CATTCAGAAGCAAGGGCAATAA  TTCATTCTCTGAACCGGAATCA  TGTTCTGATGTACGAGTGTTCA  TTGTTTTCAGAGCGACTTAAGC  CAGAAACCTGGAGAGTTTGTTG  CAAAATTTACTGCCTCACCACA  TTTGGGTCTATCAGGTTCTTCC  GTTTATCTCGCGAGATTGGAAC  GCAAGAGAAGCAATAAGAGCTC  ACCAACTCCATTTCAATTCGTG  CGGTTCTAGCTAATTGATGTGC  GATGGACGGATCAACCAAAAAT  TCTGACAGATGACACATAGACG  AATGTGAAGTCAGACCCAGATT  ACAGAAAAACCGTCTTCTTTCG  GGCATCAATTAATCACTGACCC  GACCAAATGCGCGATTAGAG  GATCTTGCAGATGCCGAATG  AATGTTTGTGTGACCATCAGTG  AAAACTTGTACATCTTGTCCGC  CTTGCGATATAGAAGGGTGTGA  CTTTGACAGGACAGATGTCTCT  CATGGAGAACTGGCATCACACCTT  CTGCGTCATTTTCTCTCTGTTGGC | 124  108  207  138  238  97  155  102  153  86  91  103  140  128  85  212  219  187  84  118 |
